# Perceived Effectiveness and Learning in Foundation Course of Medical Undergraduate Program at Patan Academy of Health Sciences: A Multi Method Study

**DOI:** 10.31729/jnma.8828

**Published:** 2024-12-31

**Authors:** Tripti Shakya, Babu Raja Maharjan, Shital Bhandary, Balakrishnan M Acharya, Paban Sharma, Rajesh Nath Gongal

**Affiliations:** 1 Department of Anatomy, Patan Academy of Health Sciences, Lagankhel, Lalitpur, Nepal; 2 Department of Biochemistry, Patan Academy of Health Sciences, Lagankhel, Lalitpur, Nepal; 3 Department of Community Health Sciences, Patan Academy of Health Sciences, Lagankhel, Lalitpur, Nepal; 4 Department of Orthopaedics, Patan Academy of Health Sciences, Lagankhel, Lalitpur, Nepal; 5 Department of Gynaecology and obstetrics, Patan Academy of Health Sciences, Lagankhel, Lalitpur, Nepal; 6 Department of Surgery, Patan Academy of Health Sciences, Lagankhel, Lalitpur, Nepal

**Keywords:** *evaluation*, *foundation course*, *Kirkpatrick model*, *medical students*, *Nepal*

## Abstract

**Introduction:**

Foundation course was introduced in medical undergraduate curriculum of Patan Academy of Health Sciences, to provide students with essential knowledge and skills for their pursuit of learning in their medical undergraduate program. This study aimed to measure perceived effectiveness of the foundation course of medical students.

**Methods:**

A multi method study was conducted among students completing the foundation course at a medical college in Nepal. The perceived effectiveness of foundation course was evaluated by applying three levels of Kirkpatrick's model i.e. reaction (satisfaction), learning and behavior. Quantitative data is presented as frequency, percentage, mean±standard deviation. Qualitative data was analyzed by coding and categorized into different themes and subthemes. Ethical approval was taken from Instutional Review Committee (Reference number: bss 2207081655).

**Results:**

A majority of students perceived the foundation course as a memorable experience that helped them to familiarize to new college environment. Students felt that it oriented them to the newer teaching learning and assessment system of the medical college. Assessment scores showed that students had significant learning in foundation course which students perceived applicable in basic and clinical science years.

**Conclusions:**

Foundation course learning has been very beneficial and applicable to the students in their medical studies. This course has helped students to get oriented to a new education system and learning environment.

## INTRODUCTION

There is a significant shift in medical education from teacher-centered to student-centered learning with emphasis on skills development, medical ethics, and better doctor-patient relationships.^1^ The teaching/learning environment and learning method in Bachelor of Medicine, Bachelor of Surgery (MBBS) are quite distinct from 10+2 (Intermediate Level).^23^ Patan Academy of Health Sciences (PAHS) has adopted Problem Based Learning (PBL) as main teaching/learning methodology in Basic Sciences and Clinical Presentation (CP) in Clinical Sciences. Additionally, community-based learning in PAHS envisions training medical doctors with competencies to work in rural Nepal.^4^ A smooth transition by orienting students to new teaching/learning methods can be beneficial.

Hence, a foundation course (FC) of eight weeks was introduced at the beginning of MBBS in 2018.

India and Pakistan have also introduced FC.^5,6^ This course is beneficial to students.^3,7^ The foundation course is available only at PAHS amongst the medical schools in Nepal. However, evidence is lacking to prove its effectiveness in our context. Therefore, this study aimed to measure the perceived effectiveness of foundation coursebased on students' satisfaction, learning, and transfer of knowledge and skills.

## METHODS

An observational cross-sectional study was conducted among the students who have completed foundation course at Patan Academy of Health Sciences, Lagankhel, Lalitpur, Nepal. Foundation course was introduced in curriculum of PAHS since 2018. Foundation course is implemented at the beginning of MBBS course for students to get acclimatized to new college environment. So far foundation course is implemented only at PAHS in Nepal. This course is developed to provide a strong foundation, in terms of required knowledge and skills to assist students to become a better doctor. This course aims to provide a basic knowledge and skills on medical humanities, medical informatics, clinical knowledge and skills, ethics, scientific communication, language of medicine and community health sciences. During this course, students have one day of old age home visit. All 259 medical students intake in year 2018 (8^th^ batch), 2019 (9^th^ batch), 2020 (10^th^ batch) and 2021 (11^th^ batch) were the total population for the study. The numbers of students from first (11^th^ batch), second (10^th^ batch), third (9^th^ batch) and fourth (8^th^ batch) year were 67, 74, 56 and 62 respectively. Students who were absent on the day of data collection and those who were involved in the pretest were excluded from the study.

The Kirkpatrick evaluation model is a widely used evaluation method that includes four levels: reaction (satisfaction), learning, behavior (transfer of knowledge and skill), and results.^8,9^ The course was evaluated based on the first three levels of Kirkpatrick's model. Questionnaires consisting of both qualitative and quantitative components were constructed based on the curricular objectives of the FC.

For evaluating reaction, students' satisfaction on FC was assessed among all four batches of students using a closed ended questionnaire. It consisted of seven general questions about FC using a four point Likert scale (strongly disagree, disagree, agree, strongly agree).

For evaluating learning, the exam scores of all four batches of medical students were used. Exams were taken at the end of FC for each batch. The purpose of the exam was to facilitate learning and orient students to the examination system of PAHS. It included multiple choice questions (MCQs), problem based questions (PBQs), Objective Structured Clinical Examination (OSCE) and viva. These are the standard components of examinations for medical undergraduates at PAHS. With permission from Dean, School of Medicine, anonymized results of students were obtained from the exam section that only contained students' score range (≤40%, 40-60%, 60-80% and 80-100%) for both the theory and practical exam.

For evaluating behavior, a pre-validated questionnaire with 13 questions was used asking students whether the learned content of FC was applicable in basic sciences (students from second (50), third (53) and fourth year (50) and clinical sciences (students from third and fourth year). The score for each question ranged from one to ten scales with one being the least applicable and ten being the most applicable. In the qualitative component of the study, open ended questions regarding overall strength of FC and suggestions from the students were asked.

For content validation, the questionnaire was discussed among members of research team. To test the reliability of questionnaire, pretest was carried out among nine students with three students each from first, second and third year. Their responses were recorded and questionnaire was modified as per the feedback provided by these students.

Study was conducted after receiving ethical approval from Institutional Review Committee, PAHS (Reference number: bss 2207081655). Before collecting data, the purpose of the study was explained to students and written consent was taken. It took around 15-20 minutes to fill up the questionnaire. Collected data was entered in excel and then analyzed using IBM SPSS Statistics for Windows, version 20 (IBM Corp., Armonk, N.Y., USA). Quantitative data was presented as frequency, percentage, mean±standard deviation. Qualitative data from open ended questionnaire was analyzed using Braun and Clarke's thematic analysis by coding and categorizing into different themes and subthemes.^10^

## RESULTS

Among total of 259 medical students, 207 (79.92%) students submitted a completed questionnaire. Out of 207 students, 50 (24.15%) students were from 8^th^ batch, 53 (25.60%) from 9^th^ batch, 50 (24.15%) from 10^th^ batch and 54 (26.10%) students from 11^th^ batches were included in the study. Among them, 126 (60.90%) were male and 81 (39.10%) were female. Ages of the students ranged from 18-32 years with 192 (92.75%) of the students in age group of 20-24 years.

Out of all students, 205 (99.03%) of students perceived that the FC was effective and had imparted a memorable learning experience, 204 (98.55 %) of students agreed that this course helped them to familiarize with the new environment. A total of 192 (92.75%) students agreed that this course should be introduced in MBBS program. Likewise, 190 (91.79%) medical students were in agreement that two months duration for the course is adequate (Table 1). However, in response to the open ended questionnaire, 24 (11.59%) of students suggested that duration of FC needed to be reduced (Table 2).

In response to open ended questions on the strength of FC, 139 (67.15%) students mentioned that this course helped students in familiarization and adaptation to new learning environment. There were 27 (13.04%) students who expressed that learning in foundation corse was fun, study period was tension free and boosted their enthusiasm for medical school.

A total of 83 (40.09%) students felt that it oriented them to the newer teaching/learning system and exam assessment system of PAHS. Forty-six (22.22%) students also expressed that the content covered in the various disciplines of the FC were helpful in the subsequent medical curriculum (Table 2).

**Table 1:** Satisfaction of medical students in foundation course (n=207).

SN	Items	Strongly Disagree N (%)	Disagree N (%)	Agree N (%)	Strongly agree N (%)
1	Foundation Course was a memorable experience.	-	2 (0.97)	72 (34.78)	133 (64.25)
2	Foundation Course must be introduced in MBBS program.	3 (1.45)	12 (5.8)	92 (44.44)	100 (48.31)
3	Foundation Course helps in familiarization to the new environment.	0 (0)	3 (1.45)	66 (31.88)	138 (66.67)
4	Two months duration of Foundation Course is adequate	6 (2.9)	11 (5.31)	99 (47.83)	91 (43.96)
5	I found PBL as a more effective teaching-learning method.	1 (0.48)	7 (3.38)	52 (25.12)	147 (71.01)
6	Foundation Course helped me to become self-directed learner.	1 (0.48)	28 (13.53)	97 (46.86)	81 (39.13)
7	I don't find this course effective.	114 (55.07)	76 (36.71)	13 (6.28)	4 (1.93)

**Table 2:** Theme, subtheme and codes obtained from open ended questionnaire regarding perceived effectiveness of foundation course among medical students (n=207).

Theme	Subtheme (no. of students)	Code
Strength	Adaptation to new environment (139)	Adaptation to new environment, familiarize to new learning environment, bridge between +2 and medical study, introduction to basic concept of medical science, sensitization toward MBBS course, acts as a gateway/transition course into basic sciences, provide ample time to transit students from 10+2 to medicine,ease student's fears and discomfort of a new learning environment, introduction to clinical aspect of medicine,give us idea how medical field is,time without tension, time to relax after joining medical school, it made each of us on common level, learn basic concept and skill that prepare us for basic science.
	Teaching learning (TL) method (83)	Orient to TL system of PAHS, self-directed learning, learn PBL system, orient to exam assessment system, interactive classes, encourage students to participate in active discussion, interactive sessions, practical demonstration and act was helpful.
	Safe/fun learning (27)	Memorable experience, create a lot of memory to cherish in future, creative learning, interaction with friends, group work during FC makes us social and comfortable, help enter medical schools with great enthusiasm, study without pressure, time without tension.
	Soft skills (18)	Effective feedback, increase confidence, anger management, built personality, cope us in difficult situation, helped in critical thinking, improve basic skill, understand lifelong skills, leadership skill, teaches us what are the hurdles that can be encountered going forward.
	Communication skill (64)	Learnt communication skills, able to speak confidently among friends, communication with patient.
Suggestion	Duration: Increase (26), Decrease (24)	Duration not enough, duration need to be reduced, decrease two hour lectures.
	Addition of content (51)	Basic of basic science disciplines, introduction of physiology, pathology and pharmacology, basic of chemistry and physics, English classes, addition of some portion of principle of human biology (PHB-1), introduction of SPSS, more biostatistics session, proper test to know the level of learning of students, research writing, addition of many lectures, basic skill with assignment, add basic terminology applicable in medical science, more clinical contents, mentor assignment, PBL case involving basic science topic, stress management sessions and field works.
	Continuation of the course (49)	Must be continued, must be included in other medical college as well, nothing to improve.
	Self-study (20)	Self-study sessions need to be reduce, extracurricular activities instead of self-study

Regarding strength of this course, students expressed: *"FC was like a healthy noncompetitive period filled with a lot of learning which is not found in textbook. It sensitizes us to the new environment of PAHS and its motto & principles. It also helped us to connect with our friends. The weekly group activities were the best part of foundation course.* I *really cherish the moments when* I *remember it. " (MS 46)*

*"Coming from 10+2 to medical school can be very intimidating but foundation course provided us time to accustom to study methodology and to manage time. It helped to build team spirit, leadership qualities, speaking and presentation skills."(MS 90)*

*"It is a pride of PAHS, so unique from other medical institute. It helps to make capable medical students with a sound social orientation and emotional abilities who can experience how to handle ups and downs further in medical carreer. "(MS 82)*

*"Everyone enrolling medical school must at their starting should have this soothing experience. It's worth the time, memories, learning and process of educating ourselves in a unique way. "(MS 122)*

*"It was one of the best part of being in PAHS. The bond that we built with faculties and friends was really nice." (MS 80)*

*"Foundation course makes a person a real human being not just a mechanical professionals." (MS 110)*

*"It was the great platform and I feel proud that our institute has taken initiative of conducting those extra efforts which is so positively impactful." (MS 86).*

One hundred and ninty-nine (96.13%) of students found problem-based learning (PBL) as an effective teaching learning method (Table1, 2). Students also expressed that they have learnt about the process of PBL which is a major teaching learning methodology in basic science years. Regarding PBL sessions students also stated:

"PBL was introduced which help to be more comfortable and confident in fulfilling process of PBL in basic science. It helped to be more confident to speak in front of class" (MS 138)

"Introduction of PBL session in FC personally helped me a lot in developing my communication, self directed learning and group skills." (MS 189).

For the measurement of student's learning during FC, examination score range was used. All the students passed the FC exams with a majority of students scoring more than 60% in both the theory and practical exam (Table 3).

Regarding the applicability of knowledge and skill learned in FC 153 students provided complete questionnaire [year second (50), third (53) and fourth (50)] related to applicability of FC in basic science years while 103 students provided completed questionnaire related to applicability of FC in clinical sciences [year third (53) and fourth (50). The mean score as rated by students on applicability of learning of effective communication with patients as 8.25±1.53 in basic science year and 8.47±1.23 out of 10 in clinical science. (Table 4).

The learnings of all of disciplines in FC were well appreciated and found to be applicable later in basic and clinical science years. Students agreed that the learning in Medical humanities (MH) has helped them to understand the concept of illness, disability, dying and to be sensitive to socioeconomic and cultural issues while taking care of patients. Similarly, students agreed that learning in introduction to clinical medicine (ICM) was applicable in making effective communication with the patient.

**Table 3:** Learning among all batches of medical students who have completed foundation course (n=207).

Intake year of students	Score range in theory (n)			Score range in Practical (n)		
	40-60%	60-80%	80-100%	40-60%	60-80%	80-100%
2018	6 (9.23)	52 (80)	7 (10.77)	6 (9.23)	44 (67.69)	15 (23.08)
2019	8 (12.31)	51 (78.46)	6 (9.23)	12 (18.46)	44 (67.69)	9 (13.85)
2020	9 (13.85)	51 (78.46)	5 (7.69)	3 (4.62)	35 (53.85)	27 (41.54)
2021	2 (3.08)	40 (61.54)	23 (35.38)	1 (1.54)	55 (84.62)	9 (13.85)

**Table 4:** Transfer of knowledge and skill learnt from foundation course in basic and clinical science years (n=207).

SN	Items	In Basic Science (n=153) Mean±SD	In Clinical Science (n=103) Mean±SD
1	I applied the presentation skill that was learnt in Scientific Communication.	8.02±1.60	8.05±1.65
2	I applied the power point slide preparation skill that was learnt in Scientific Communication.	8.12±1.46	8.06±1.52
3	I applied the data summarizing skill that was learnt in Scientific Communication.	7.01±1.90	7.39±1.74
4	I became sensitive to illness, disability and dying.	8.16±1.46	8.38±1.32
5	I became sensitive to socioeconomic and cultural issues while taking care of the patient.	8.17±1.46	8.40±1.20
6	I applied the learning of effective communication with patient.	8.25±1.53	8.47±1.23
7	I applied the theories of bioethics in taking consent.	8.16±1.53	8.14±1.57
8	I applied the learning of First Aid management.	6.57±2.47	7.61±2.06
9	I applied the learning of basic combining forms in medical terminologies construct.	8.07±1.83	7.86±1.88
10	I applied the Microsoft Word Formatting skill	7.65 ±1.92	7.51±1.89
11	I applied the Microsoft Excel in preparing graphs.	7.99±1.78	7.48±1.75
12	I applied the Microsoft Excel in calculation.	7.91±1.80	7.34±1.88
13	I applied the learning of National Health System.	7.25±2.00	7.42±1.93

Regarding suggestions to FC, 40 students suggested the need for more practical sessions/group works 32 Students suggested introducing basics of science subjects like Physiology, Pharmacology, Pathology, biocehemistry, stress management sessions, research writing as well as assignment of mentor. They were keen that this course be continued at PAHS and be included in other medical colleges of Nepal. Students perceive that they have too many self-study sessions that need to be reduced or be replaced by extracurricular activities (Table 2). Suggestions of students' regarding this course:

*"Introduction of Basic idea regarding pharmacology, physiology and pathology would have made it much better." (MS 45)*

*"Self-study time should be decreased rather there should be extracurricular activities emphasized by college itself." (MS 20)*

*"English language still remains to be very big barrier so classes aiding to help students could be useful."(MS 60)*

## DISCUSSION

The most of students appreciated the provision of FC in the beginning of medical curriculum. Other studies have also shown a high satisfaction among the students with the course.^3,11,12^ Students found that the generic learning/skill such as communication skill, history taking skill, computer skill were applicable later in basic science and clinical sciences. They also reflected that such learning has enabled them to understand concepts which otherwise could have been difficult to grasp in subsequent years because of other learning priorities. Similar findings were reported that showed students perceived FC as an excellent opportunity to acquire basic knowledge, attitude, and skills required for subsequent phases in MBBS course and later in their medical career.^13,14^ P Students perceived that FC acted as a bridge between 10+2 and medical study which helped them to get familiarized with the new learning environment. This finding is consistent with the study elsewhere where they have also found that FC has helped students to adapt to new learning environment.^15^ In congruence to earlier studies, majority of students agreed that PBL is effective teaching learning method and it had helped them to be aself-directed learner.^16-18^

Students preferred more interactive sessions that includes practical sessions and group work than didactic theoretical session although there is already a planned group activities in the delivery of the curriculum. In the implementation of the FC content, faculty have used mixed approach in delivering the content constituting both didactic session to provided theoretical background and interactive group/practical work to facilitate learning. This approach indeed is in accordance to the merit of group-activities that bring out creativity and inculcate team spirit among students for developing a problem-solving mindset^19^ Hence, it would be beneficial if more group activities could be incorporated wherever feasible in the delivery of the content.

Another suggestion by students was a necessity of having stress management sessions which was consistent to the finding of another study where student felt a need of such sessions on a regular basis.^20^ Adequate self-study sessions had been ensured in academic scheduleto promote rapport building among the friends and adapt to new environment. Students also reflected that provision of such self-study time has enable them to know each other well. However, some students also perceived that they had too many self-study sessions and suggested to reduce it or alternatively replace it by extracurricular activities that will help them to pursue their hobbies that can act as a relaxant in their hectic academic schedule and help to improve their academic performance.

The findings of the study showed that FC laid the foundation for a better learning of medical undergraduate program. The students have also perceived that FC is relevant and applicable in their subsequent busy medical study. Therefore, FC undertaken at PAHS has been able to provide basic communication and history taking skills; develop understanding towards disability, disease, death, dying and ethical values; application of basic computer skills, understanding approaches in scientific writing, health system and formation of medical terminologies. Earlier studies at PAHS on Medical Humanities had shown that it increased the Empathy scores of students and majority of students agreed that it helped them to understand the caring roles of a doctor.^21,22^ Other study also resonates similar advantage of FC for asmooth transition into the rigorous medical study for the students coming directly from schools.^23^ Although MCI in India has made a month long FC mandatory,^5^ in Nepal the course in not mandatory and is not implemented in other medical school besides PAHS. Although the basis of drawing these inferences are limited to the perception of students, inclusion of other stakeholder's (faculty and academic administrators) perception will further augment the evidence. However, the findings of this study provide reasonable ground for Medical Education Commission to consider mandatory implementation of FC in undergraduate medical Program across Nepal.

## CONCLUSIONS

The FC has provided an opportunity to get oriented with new learning environment, new teaching learning methods and new assessment system. Students perceived that it has primed them in a fun environment for the learning of the subsequent medical curriculum. They found learning in different disciplines of FC were applicable in basic and clinical science that helps them to be a better doctor.
